# Ultrasound classification-guided minimally invasive rotary cutting in granulomatous lobular mastitis

**DOI:** 10.1186/s12905-020-01118-y

**Published:** 2020-11-16

**Authors:** Hongye Liao, Jujiang Guo, Xuming Chen, Zhipeng Hua, Juli Lin, Yiyin Weng

**Affiliations:** Department of Breast Surgery, Women and Children’s Hospital, School of Medicine, Xiamen University, No. 10 Zhenhai Road, Xiamen, 361000 Fujian China

**Keywords:** Mammotome minimally invasive rotary cutting technology, Granulomatous lobular mastitis (GLM), Ultrasonography classification, Esthetic outcomes

## Abstract

**Background:**

To summarize the clinical experience of ultrasound-guided minimally invasive surgery for granulomatous lobular mastitis (GLM), and explore the feasibility of this technique for treating GLM.

**Methods:**

This retrospective study reviewed the clinical features and treatment outcome of 30 patients who were diagnosed pathologically as GLM from 2016.3 to 2019.5 in the Department of Breast Surgery, Women's and Children's Hospital, Xiamen University. These patients weretreated with ultrasound-guided Mammotome minimally invasive surgery, and we tried to classified the lesion into four distinct patterns (diffuse abscess mixed type, sheet hypoechoic type, localized abscess type, localized hypoechoic mass type) according to the sonographic findings and clinical symptoms to find out if these patterns correlated with treatment and recurrence rate.

**Results:**

After a median follow-up of 12 months on average (4–42 months), 26 cases (86.7%) were cured without acute or chronic complications such as disseminated inflammation and bleeding. Post-operative bleeding occurred in 1 case, and 3 cases (10.00%) relapsed. The ultrasound classification had 0 cases of diffuse abscess mixed type, 17 cases (56.7%) of sheet hypoechoic type, 9 cases (30%) of localized abscess type, and 4 cases (13.3%) of localized hypoechoic mass type. All 3 recurrent cases were sheet hypoechoic type, which were cured after another open surgical resection and showed no recurrence during an average follow-up of 20 months (11–40 months).

**Conclusions:**

In treating GLM patients with minimally invasive rotary cutting, ultrasound classification helps to select suitable patients, especially those with localized abscess and localized hypoechoic mass types with low recurrence rate, which is one of the safe and effective treatment methods.

## Background

**Fig. 1:**
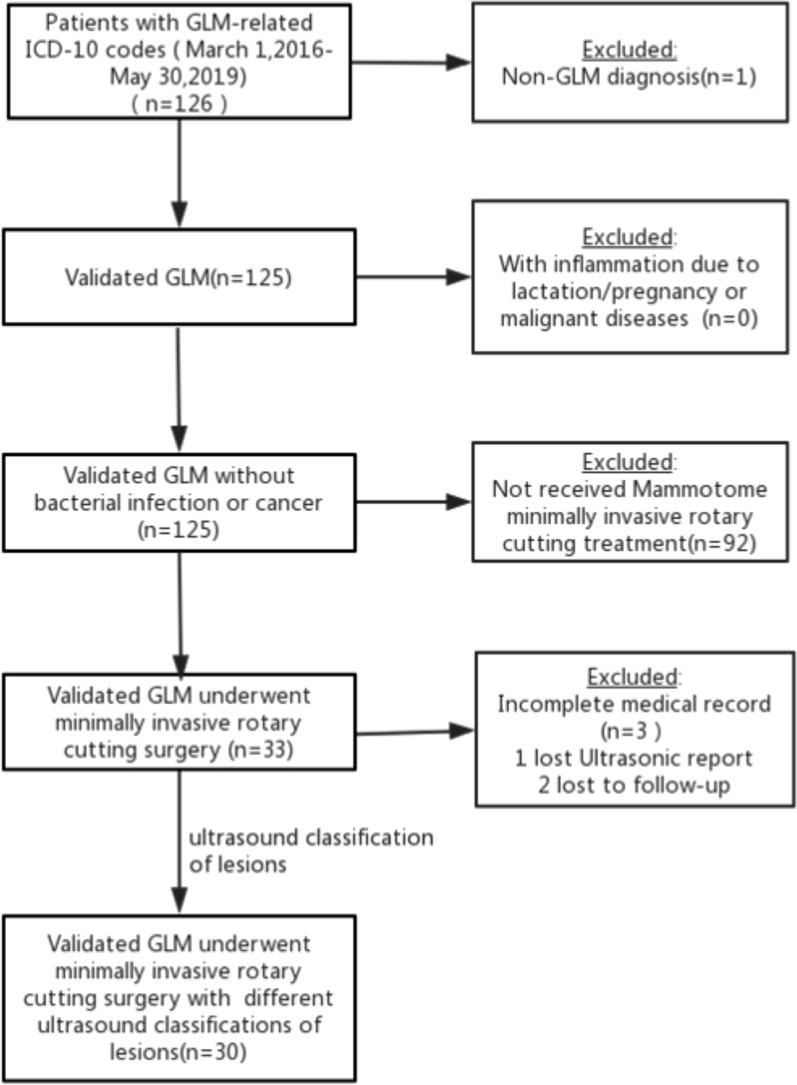
Flowchart for selecting patients undergoing minimally invasive surgery

Granulomatous lobular mastitis (GLM) is a chronic non-specific inflammatory disease, limited to the lobules of the breast tissue, with the main pathological characteristic of necrotizing granuloma [1]. It is a rare, benign condition of the breast with poorly understood etiology, unpredictable duration, and lack of consensus about optimal treatment. It mainly occurs in post-pregnant and non-lactating young and middle-aged women. The common clinical manifestations include non-cyclic breast pain, nipple discharge, inverted nipple, breast lumps, non-lactating breast abscess, cutaneous fistula, etc. Similarities with other diseases clinically and radiographically can lead to diagnostic and therapeutic delays. The optimal management of GLM is not yet defined. Corticosteroid combined surgery is used as the primary treatment for GLM, but it has high post-operative recurrence rate and also affects patient’s quality of life due to post-operative breast deformity. These challenges need to be addressed urgently. Yaghan [2] first described a classification system for IGM based on clinical grounds. Among the study group, IGM could be classified into 4 distinct patterns which are correlated with treatment, recurrence rate. However, the lesions are normally multiple and distributed, and clinical palpation findings cannot always be consistent with the scope of the disease. Ultrasound examination is helpful to further clarify the presence of abscesses and the scope of the lesions, ultimately, to provides therapeutic clues. Referring to the preceding classification and the combined experience of our department, we categorized the GLM into four groups. In this study, we described a minimally invasive treatment for GLM with low recurrence rate and good post-operative breast appearance. We selected 30 cases under the ultrasound classification, and attempted to use minimally invasive rotary cutting technology to treat GLM patients, and some positive results have been achieved. We retrospectively analyzed the clinical features and prognosis of these patients. The aim is to find out if ultrasound classification can help to select suitable patients to treat with ultrasound-guided Mammotome minimally invasive surgery. Flowchart for selecting patients undergoing minimally invasive surgery (Fig. 1).

## Method

### Patients

We reviewed the clinical data of patients who were pathologically diagnosed with GLM and were undergoing Mammotome minimally invasive rotary cutting surgery in the Women and Children's Hospital, Xiamen University between March 2016 and May 2019.

Inclusion criteria: female; patients who were clinically diagnosed and pathologically confirmed with GLM; and underwent Mammotome minimally invasive rotary cutting treatment.

Exclusion criteria: patients with inflammation due to lactation or pregnancy; patients with malignant diseases; other possible causes of mammary inflammation or granulomatous changes.

### Ultrasound classification of lesions

**Fig. 2:**
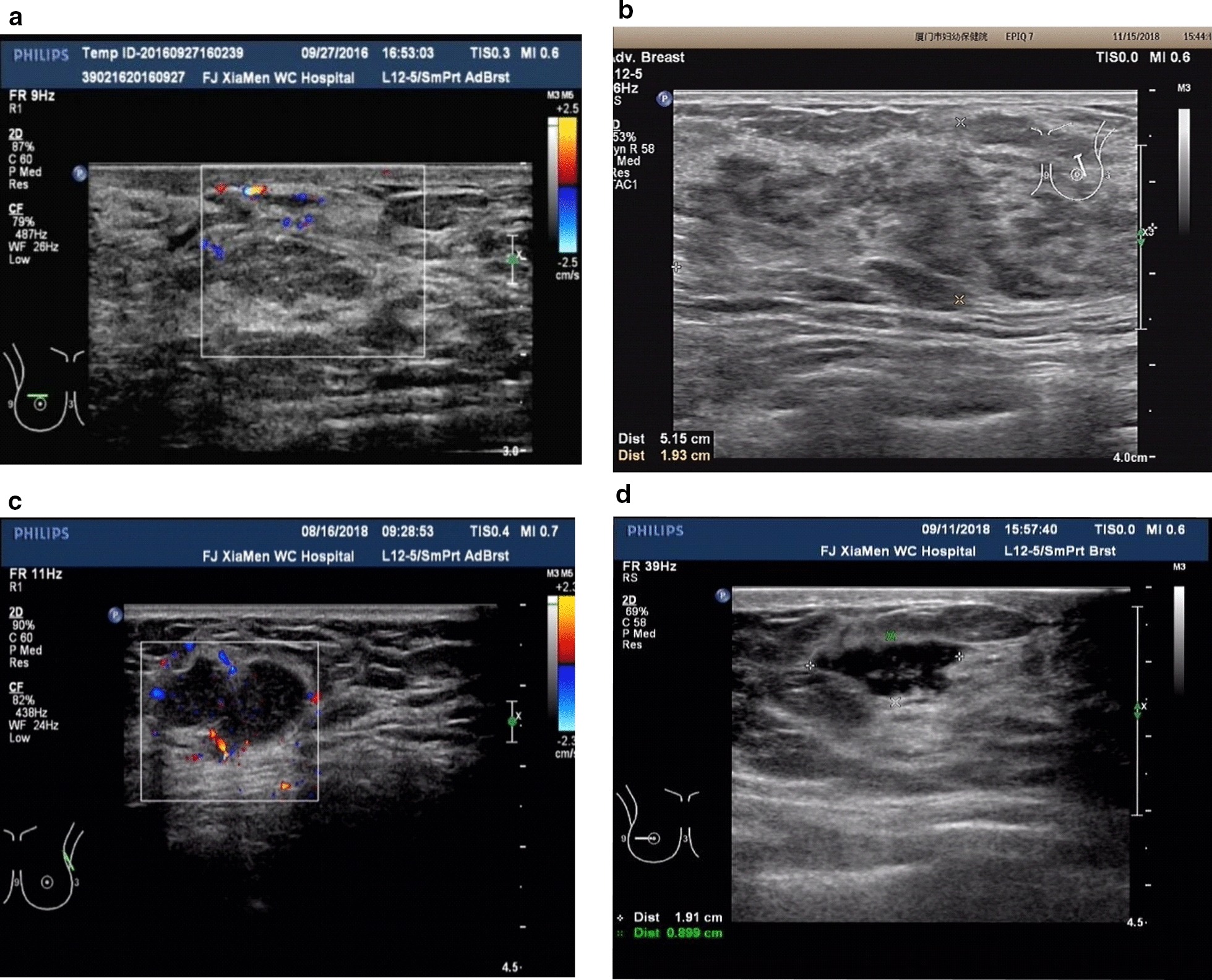
Analysis of the results of ultrasound classification of lesions. **a** A mixed type of diffuse abscess. Large patchy hypoechoic or mixed echo areas scattered; glands and multiple abscess cavities under the skin form tunnels; **b** a sheet hypoechoic type. Sheet hypoechoic area showed unclear borders, disordered structure, and no abscess cavity; **c** a localized abscess type. There were multiple abscesses with clear borders; **d** a localized hypoechoic mass type. The echo was uneven and irregular, but with clear borders

As described in the literature, ultra-sonographic findings of granulomatous lobular mastitis are nonspecific and are occasionally interpreted as malignant [3, 4]. Yaghan [2] first proposed a classification system for IGM that provides therapeutic clues and helps to predict recurrence. Referring to this classification and the combined the experience of our department, the ultrasound classification of the lesion can be categorized as: diffuse type (large patchy hypoechoic or mixed echo area scattered in the lobules; often existing across multiple quadrants without obvious borders; no display of normal glands in the lesion area, and the echo is significantly lower than that of normal glands; many low and weak abscess cavities in the glands with multiple irregular sinus tracts, which can be penetrated and can further invade the skin to form sinus tracts with rich blood supply), sheet hypoechoic type (confined in one quadrant, mainly characterized by patchy hypoechoic areas with or without abscesses inside, and with unclear borders), localized abscess type (single or multifocal abscesses with clear borders) and localized hypoechoic mass type (hypoechoic nodules with uniform or uneven internal echo, with clear borders) (Fig. 2).

### Indication for mammography

All patients were recommended for mammography except for pregnant women and patients who refused.

### Therapeutic methods

Glucocorticoid therapy: methylprednisolone tablets or equivalent dose of prednisone tablets were used. The initial dosage of methylprednisolone was 20 mg/day, which was reduced to 16, 12, 8, 4 mg/day every 1–2 weeks until the drug was stopped. Surgery was performed when there was no obvious acute inflammation and the mass remained stable and localized. Glucocorticoid doses were gradually reduced after surgery. Direct surgery was performed when the diameter of the newly diagnosed lesion was less than 2 cm or there were contraindications for glucocorticoid use (such as active peptic ulcer, poorly controlled hypertension, psychosis, fungal or viral infection) or the patient refused to use glucocorticoid.

**Fig. 3:**
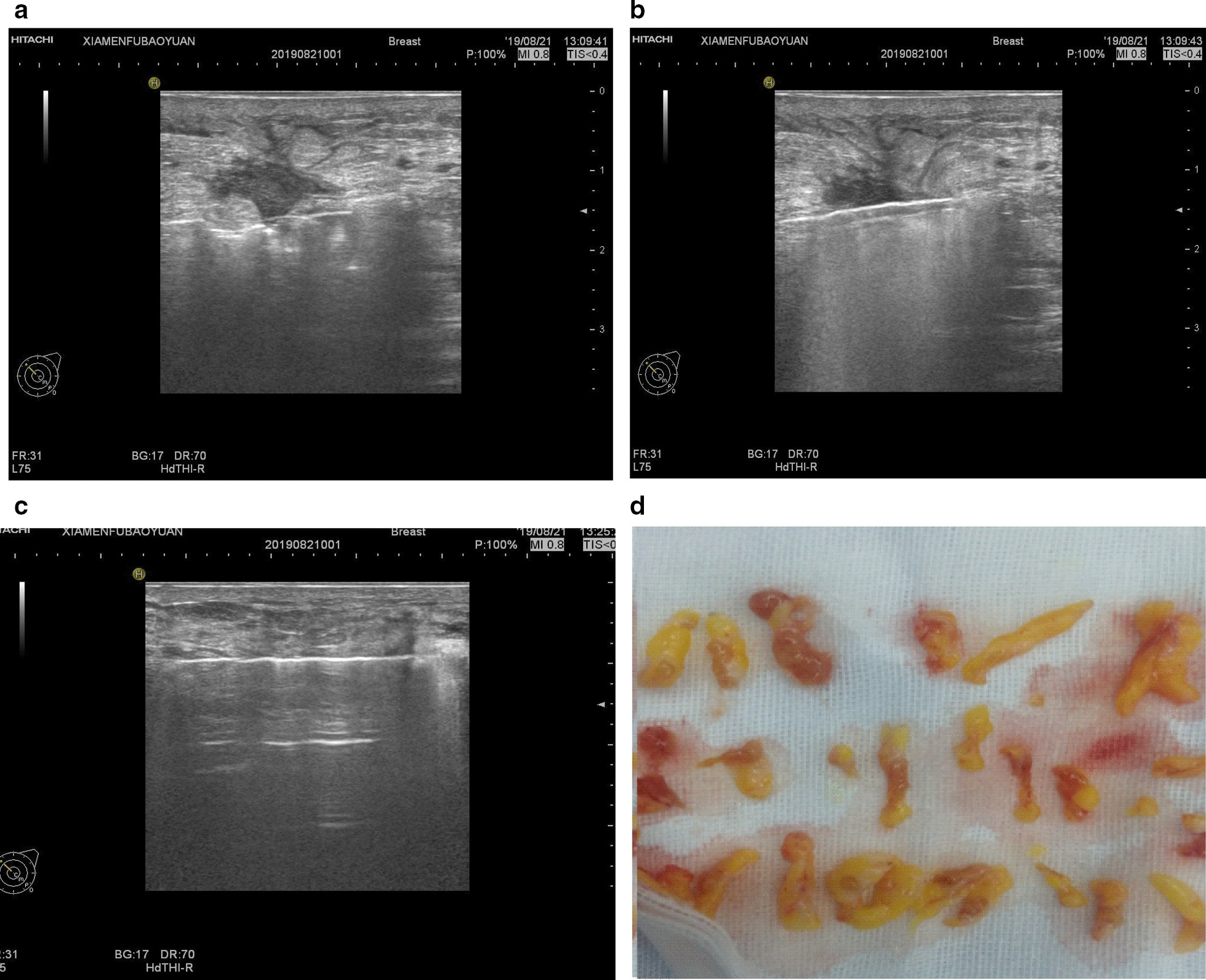
Ultrasound-guided minimally invasive rotary surgery procedure. **a** Under ultrasound guidance, the probe was punctured under the lesion with the groove facing the lesion; **b** During the resection, the ultrasound probe was kept parallel to the probe cutting groove. Under the ultrasound guidance, the angle of the cutting groove was switched in a fan shape, and the probe, target lesion and the cutting groove was kept in the same plane; **c** After the resection, the cutting groove was closed and the probe was withdrawn; **d** No obvious border between the lesion and the normal tissues was observed in the resected gross biopsy, and the dilated catheter was filled with erosive and necrotic tissues

**Fig. 4:**
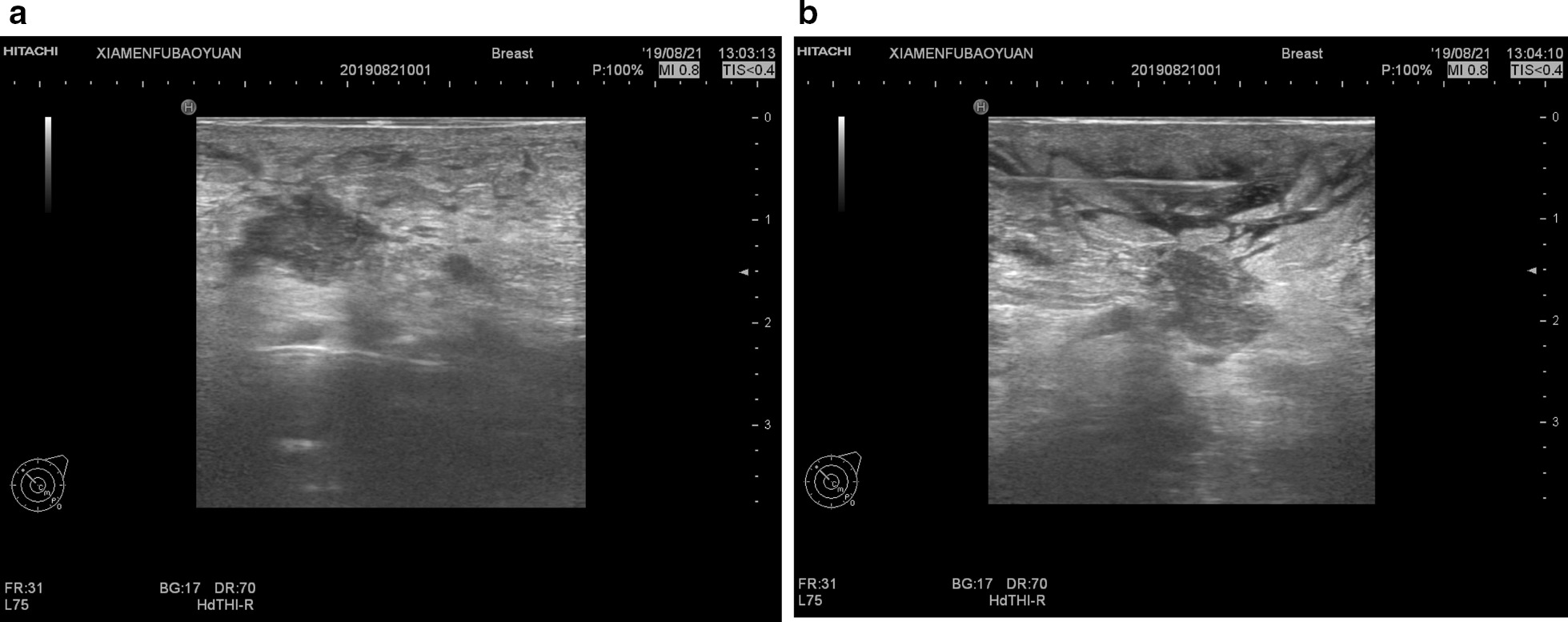
Local infiltration of anesthesia guided by ultrasound. **a** Anesthesia in the posterior breast space; **b** Anesthesia in the subcutaneous tissue space

Minimally invasive rotary cutting surgery: Surgery was performed when there was no obvious acute inflammation and the mass remained stable and localized after glucocorticoid therapy, or when the diameter of the newly diagnosed lesion was less than 2 cm, or there were contraindications for glucocorticoid use (such as active peptic ulcer, poorly controlled hypertension, psychosis, fungal or viral infection) or the patient refused to use glucocorticoid,

Mammotome Minimally Invasive Rotary Surgery System produced by Johnson & Johnson (sCM23; 8G sampling gun) and a digital color Doppler ultrasound diagnostic apparatus (MyLab™; 6–18.0 MHz in probe frequency) were used. Local anesthesia was given using a mixture of 20 ml of 2% lidocaine, 250 ml of 0.9% sodium chloride and 1 ml of epinephrine, with the final concentration of epinephrine being 4 μg/ml. We selected the submammary fold as the incision site, punctured the minimally invasive rotary cutter under the lesion and removed the lesion under the guidance of ultrasound. The scope of lesion resection consisted of palpable lesions, visible necrotic granulomatous tissues, inflammatory lesions including galactosis and dilated catheters and skin damages, and any suspicious hypoechoic nodules under intraoperative ultrasound, as well as dilated catheters and normal tissues within 0.5 cm of the surrounding area. After the withdrawal of the rotary cutter, diluted iodine and normal saline were administered through the needle tract until the flushing fluid was clear. After the operation, we placed a drainage tube through the needle tract to connect with a negative pressure ball for drainage. Appropriate compression bandage was applied for 48 h after operation. We observed the nature of the drainage tube every day. The tube was extubated when the drainage fluid was less than 5 ml for two consecutive days. The procedure of minimally invasive resection is shown in Figs. 3 and 4.

### Criteria for evaluating treatment

Healing refers to when no lesion was found by ultrasound from the diagnosis to surgical wound healing; recurrence refers to the occurrence of the same symptoms diagnosed as GLM in the affected breast after the disappearance of the initial clinical symptoms or after resection.

### Statistical analyses

All data processing and statistical analyses were performed using SPSS 20.0. The measurement data were represented by (x ± s), and the counting data were represented by (n, %).

## Results

### General baseline characteristics

30 cases met the inclusion criteria, of which, all were female, with an average age of 32 years (26–43 years). All cases had childbearing history, and went to the doctor with breast lumps and without inverted nipples, no systemic manifestations such as fever, night sweats and fatigue, and no extramammary symptoms such as erythema nodosum and arthritis. 29 cases were unilateral and 1 case was bilateral. The location of the disease was different with all quadrants visible. The average maximum diameter of the lesion was 3.43 cm (1–6 cm) 0.27 cases (27/30, 90%) had pain, 3 cases had no clinical symptoms and tumors were found by opportunistic ultrasound screening. 21 cases (21/30, 70%) were treated with glucocorticoids before surgery, of which 20 cases were hormone sensitive (the tumor was significantly reduced after treatment), and 1 case had no significant change in tumor size after treatment (1/21, 4.76%). 3 cases received drainage in other medical institutions before. (Table 1).

**Table 1:** General information of patients

Items	Cases (%)
Age (years)	
≤ 32	22 (73.3)
> 32	8 (26.7)
The diameter of lesion (cm)	
≤ 3	12 (40)
> 3	18 (60)
Pain	
Yes	27 (90)
No	3 (10)
Duration of disease (months)	
≤ 2	18 (60)
> 2	12 (40)
Hormone therapy	
Yes	21 (70)
No	9 (30)
Effectiveness of hormone therapy	20 (95.2)

### Analysis of the results of ultrasound classification of lesions

There were 17 cases (17/30, 56.7%) of sheet hypoechoic type, 9 cases of localized abscess type (9/30, 30%) and 4 cases of localized hypoechoic mass type (4/30, 13.3%) (Table 2). 28 patients received preoperative mammography examinations before surgery except the rest two who refused. Mammography examination was non-specific, showing an asymmetric density or an ill-defined mass which can be accompanied by skin edema and thickening.

**Table 2:** Ultrasound classification of lesions and recurrence

Ultrasound classification	Cases (%)	Postoperative recurrence cases (%)
Diffuse type	0 (0)	–
Sheet hypoechoic type	17 (56.7)	3 (17.6)
Localized abscess type	9 (30)	0
Localized hypoechoic mass type	4 (13.3)	0
All	30 (100)	3 (10)

After surgery, 27 cases were placed with the drainage tube for an average of 7 days (3–20 days). After a median follow-up of 12 months on average (4–42 months), 26 cases (86.7%) were cured without complications such as disseminated inflammation or bleeding, and were satisfied with the esthetic outcomes. Post-operative bleeding occurred in 1 case, and 3 cases (10.00%) relapsed. Recurrence cases all were ‘sheet hypoechoic type’, occurred within 1 month on average (3 days–1 month) after surgery, and were cured after widely surgical resection.

## Discussion

GLM is a special kind of non-lactating mastitis, with different clinical symptoms and imaging manifestations, which can be easily confused with breast cancer and breast tuberculosis. The preoperative misdiagnosis rate can reach more than 50% [5]. The typical GLM is characterized by an acute onset, usually with a painful breast mass. The mass can increase rapidly in a short period of time if not treated appropriately in time. Localized abscesses are small and scattered first, then gradually expand and infiltrate the subcutaneous and form sinuses. Some cases are self-limited, with stable size localized masses and self-relieved pain. They are often diagnosed with painless masses, and it is difficult to distinguish them from breast cancer. In our study, 4 cases were diagnosed with painless masses and received minimally invasive surgery 2 of them were diagnosed with breast fibroadenoma before surgery. The preoperative and postoperative GLM diagnosis coincidence rate was 93.94% of all.

The optimal treatment for GLM is still controversial. Currently, Chinese experts agree that hormones should be used first to reduce the lesion, followed by surgery, which can not only remove the lesion and reduce recurrence, but also maintains the esthetic outcomes of the breast [6]. Retrospective studies indicated that the remission rate of glucocorticoid treatment varies largely, from 30 to 100%, which may be related to the type and dosage of drug, and the sensitivity of the lesion to hormones [7–10]. According to our experience, it is difficult for patients with a disease duration for more than one week to achieve a complete remission by solely relying on medication. Therefore, early diagnosis and treatment are important. Timely surgery when the lesion is stable, can increase the cure rate. Traditional surgical methods have a greater impact on the appearance of the breasts, and if it does not reach a negative margin or omit minute lesions that are usually hidden in adipose tissue during the surgery, which may cause relapse. According to literature [11], there is still a local recurrence rate of about 25% after widely insection surgery. In this study, the recurrence rate was 10%, which is a favorable outcome. Moreover, Ultrasound-guided Mammotome minimally invasive technology has a distinct advantage in removing multiple, scattered and minor lesions. With the help of ultrasonic image, it can remove all suspicious lesions, preserving uninvolved breast tissues among the lesions at the same time. Thereby, it is possible to avoid appearance damage of breast due to excessive removal of tissues. It has been reported that this technique is safe and effective in the treatment of non-lactating mastitis [12].

According to the different stages of the disease and different ultrasound images, we attempted to divide GLM into diffuse abscess mixed type, sheet hypoechoic type, localized abscess type, and localized hypoechoic mass type, different treatments were given according to the specific type. This is the very first attempt to reach a classification for IGM combined clinical grounds and ultrasonoscopy. Among them, the diffuse type is characterized by widely distributed lesions, and minimally invasive surgical incision is not suitable. The remaining three types can be treated with minimally invasive surgery when the inflammation is stabilized. In the ‘localized abscess type’ and ‘localized hypoechoic mass type’ groups, the absence of recurrence suggests that these two types might be the optimal candidates for minimally invasive surgery because they usually have a smaller lesion area than the other two types, and their border of lesion are easy to define. For ‘sheet hypoechoic type’, 3 cases relapsed. The reasons of recurrence could be: (1) The area of the lesion was wide, and small lesions were hidden in the normal-like gland tissues, which could be the cause of recurrence; (2) In 1 case, the lesion in the upper inner quadrant was resected, while the lesion recurred in the upper outer quadrant adjacent to the operated area, which might be caused by insufficient resection of the surrounding inflammatory tissues. This suggests that cases with the characteristics of unclear lesion borders and uncertain extent of the lesions, are more likely to recur after surgery. For hormone-effective cases, the timing of surgery can be appropriately delayed after resolution of acute inflammation. Moreover, any abnormal tissue identified by palpating or shown as hypoechoic area shown by ultrasound, should be removed as completely as possible. It is important to carefully check the specimen, and stop the resection until normal breast gland tissue appears. Due to the wide range of resection, acute complications such as bleeding and hematoma formation are prone to occur after surgery. In our center, there have been cases of GLM cured by drainage, but new micro-abscesses often appeared in the process of dressing change after surgery. These cases required repeated surgeries and prolonged dressing change time. The average curative time was 2.4 months. For the residual lesions that have been stabilized after drainage, minimally invasive surgery can be used to reduce the treatment time. In our study population, 3 cases received minimally invasive treatment after drainage, and all cases recovered one week after the surgery without recurrence. In the past, it was believed that minimally invasive rotary cutting surgery is more suitable for tumors within 3 cm in diameter. In our study population, 4 cases of localized abscess type and localized hypoechoic mass type with diameter more than 3 cm achieved phase I healing without recurrence, indicating that minimally invasive treatment may be as safe and effective as traditional widely resection surgery regarding larger breast tumors with a diameter of 3–6 cm. Cases with congenital inverted nipple are unsuitable for minimally invasive surgery because the inverted nipple must be corrected at the same time to reduce postoperative recurrence rate. A drainage tube should be placed through a minimally invasive incision to facilitate the healing. Prolonging the extubation time may reduce the local recurrence rate. In short, strict case selection, precise positioning under the guidance of high-resolution ultrasound, complete resection, and surgeons with rich experience of minimally invasive surgery are the keys to successful treatment.

## Conclusion

In this paper, we tried to propose a classification of IGM according to the sonographic findings and clinical symptoms to help selecting the patients for the minimally invasive treatment. In the patient cohort of our study, ultrasound guided minimally invasive cutting technology for treatment of GLM showed low recurrence rates and maintained the beauty of breast. However, the limitation of our research is obvious: It is retrospective, the sample size is too small, and treatment strategy have been performed according to clinical preference of surgeon. Thus, individual preferences may have interfered with proper treatment strategy. Further studies are needed to verify its safety and effectiveness.

## Data Availability

The datasets used and/or analysed during the current study available from the corresponding author on reasonable request.

## Abbreviation

GLM: Granulomatous lobular mastitis

## References

[1] Korkut E (2015). Granulomatous mastitis: a ten-year experience at a University Hospital. Eurasian J Med.

[2] Yaghan R (2019). A proposal of a clinically based classification for idiopathic granulomatous mastitis. Asian Pac J Cancer Prev.

[3] Arslan S (2018). Advantages of b-mode ultrasound combined with strain elastography in differentiation of idiopathic granulomatous mastitis from malignant breast lesions. Turk J Med Sci.

[4] Fazzio RT (2016). Idiopathic granulomatous mastitis: imaging update and review. Insights Imaging.

[5] Cabrera G, Medina R (2013). Idiopathic granulomatous mastitis: a benign lesion with malignant clinical-radiological characteristics. Radiologia.

[6] Erozgen F (2010). Corticosteroid treatment and timing of surgery in idiopathic granulomatous mastitis confusing with breast carcinoma. Breast Cancer Res Treat.

[7] Ma X, Min X, Yao C (2020). Different treatments for granulomatous lobular mastitis: a systematic review and meta-analysis. Breast Care (Basel).

[8] Aghajanzadeh M (2015). Granulomatous mastitis: presentations, diagnosis, treatment and outcome in 206 patients from the north of Iran. Breast.

[9] Cetin K (2019). Comparison of topical, systemic, and combined therapy with steroids on idiopathic granulomatous mastitis: a prospective randomized study. World J Surg.

[10] Kaviani A (2019). Idiopathic granulomatous mastitis: looking for the most effective therapy with the least side effects according to the severity of the disease in 374 patients in Iran. Breast J.

[11] Yau FM (2010). The surgical management of granulomatous mastitis. Ann PlastSurg.

[12] Wang Y (2020). Minimally invasive comprehensive treatment for granulomatous lobular mastitis. BMC Surg.

